# Integrated Radiomics Model Combining Diffusion Kurtosis Imaging and Dynamic Contrast‐Enhanced MRI for Predicting TERT Promoter Mutation Status in Gliomas

**DOI:** 10.1155/humu/6650170

**Published:** 2026-04-07

**Authors:** Song Gao, Shenao Zhang, Yinjiao Wang, Lang Chen, Aihong Cao, Peng Du

**Affiliations:** ^1^ Department of Radiology, The Second Affiliated Hospital of Xuzhou Medical University, Xuzhou, Jiangsu, China, xzmc.edu.cn; ^2^ Cancer Institute, Cellular Therapeutics School of Medicine, Xuzhou Medical University, Xuzhou, Jiangsu, China, xzmc.edu.cn

**Keywords:** diffusion kurtosis imaging, dynamic contrast-enhanced magnetic resonance imaging, glioma, promoter mutation, radiomics, telomerase reverse transcriptase

## Abstract

**Purpose:**

The purpose of this study is to investigate the value of a radiomics model based on diffusion kurtosis imaging (DKI) and dynamic contrast‐enhanced magnetic resonance imaging (DCE‐MRI) for preoperative prediction of telomerase reverse transcriptase (TERT) promoter mutation status in gliomas.

**Methods:**

This retrospective study included 126 patients with pathologically confirmed gliomas who underwent TERT promoter mutation testing between January 2020 and June 2025. All patients underwent preoperative multiparametric MRI including DKI and DCE‐MRI sequences. Patients were randomly divided into training (*n* = 88) and validation (*n* = 38) cohorts at a 7:3 ratio. Radiomics features were extracted from DKI parameter maps (mean kurtosis, mean diffusivity, axial kurtosis, radial kurtosis, axial diffusivity, and radial diffusivity) and DCE‐MRI parameter maps (volume transfer constant [Ktrans], extravascular extracellular volume fraction [Ve], rate constant [Kep], and plasma volume fraction [Vp]). Feature selection was performed using the least absolute shrinkage and selection operator (LASSO) regression. DKI‐based, DCE‐MRI‐based, and combined radiomics models were constructed using logistic regression. Model performance was evaluated using receiver operating characteristic (ROC) curve analysis, and comparison between models was performed using the DeLong test. Decision curve analysis was conducted to assess clinical utility.

**Results:**

Significant differences in DKI and DCE‐MRI parameters were observed between TERT promoter mutant and wild‐type gliomas (*p* < 0.05). LASSO regression selected 12 optimal features (five from DKI and seven from DCE‐MRI) for the combined model. In the training cohort, the DKI‐based model, the DCE‐MRI‐based model, and the combined model achieved areas under the curve (AUCs) of 0.847 (95% confidence interval [CI]: 0.768–0.926), 0.892 (95% CI: 0.821–0.963), and 0.961 (95% CI: 0.927–0.995), respectively. In the validation cohort, the corresponding AUCs were 0.823 (95% CI: 0.691–0.955), 0.869 (95% CI: 0.752–0.986), and 0.943 (95% CI: 0.871–1.000). The combined model demonstrated significantly superior performance compared to single‐modality models (*p* < 0.05), with sensitivity, specificity, and accuracy of 88.9%, 95.0%, and 92.1% in the validation cohort. Decision curve analysis indicated that the combined model provided greater clinical net benefit across threshold probabilities ranging from 0.15 to 0.85.

**Conclusion:**

The integrated multiparametric radiomics model combining DKI and DCE‐MRI enables noninvasive preoperative prediction of TERT promoter mutation status in gliomas with high accuracy. The combined approach demonstrates superior predictive performance and clinical utility compared to single‐modality imaging, providing valuable imaging biomarkers for molecular stratification and personalized treatment planning in glioma patients.

## 1. Introduction

Gliomas represent the most common primary malignant tumors of the adult central nervous system, accounting for more than 80% of all primary brain tumors. Their invasive growth characteristics and high recurrence rates result in poor patient prognosis [1]. In recent years, rapid advances in molecular biology have greatly promoted paradigm shifts in glioma diagnosis and treatment. The 2021 fifth edition of the World Health Organization Classification of Tumors of the Central Nervous System emphasizes the central role of molecular biomarkers in glioma diagnosis and grading, integrating histopathological features with molecular genetic alterations to establish an integrated diagnostic framework [2]. This classification system divides adult‐type diffuse gliomas into three major categories: IDH‐mutant astrocytoma, IDH‐mutant and 1p/19q‐codeleted oligodendroglioma, and IDH‐wildtype glioblastoma. For the first time, specific molecular biomarkers have been incorporated into tumor grading criteria, marking the transition of glioma diagnosis from traditional morphological classification toward precision stratification based on molecular phenotypes [3, 4]. This paradigm shift provides a more reliable biological foundation for formulating individualized treatment strategies and prognostic assessment.

Among numerous molecular biomarkers, telomerase reverse transcriptase (TERT) promoter mutations, as one of the most common molecular genetic alterations in gliomas, have increasingly prominent clinical importance. The two hotspot mutation sites, TERT promoter C228T and C250T, create novel ETS transcription factor binding sites, leading to upregulation of TERT gene expression and enhanced telomerase activity, thereby conferring unlimited proliferative capacity and immortalization to tumor cells [5]. The frequency of TERT promoter mutations varies significantly across different molecular subtypes of gliomas, with detection rates reaching up to 70% in IDH‐wildtype glioblastomas, exceeding 90% in IDH‐mutant and 1p/19q‐codeleted oligodendrogliomas, and only approximately 10% in IDH‐mutant astrocytomas [6]. More importantly, the prognostic significance of TERT promoter mutations demonstrates heterogeneity across different molecular contexts. In IDH‐wildtype glioblastomas, this mutation is associated with shorter survival and worse prognosis, whereas in certain IDH‐mutant lower grade gliomas, it may indicate relatively favorable clinical outcomes [7]. Furthermore, according to the 2021 WHO classification criteria, even in the absence of histological malignant features such as necrosis or microvascular proliferation, IDH‐wildtype diffuse astrocytomas harboring TERT promoter mutations, EGFR amplification, or Chromosome 7 gain combined with Chromosome 10 loss can be diagnosed as glioblastoma [8]. This underscores the critical value of TERT promoter mutation status detection for precise diagnosis, treatment decision‐making, and prognostic assessment of gliomas.

However, traditional TERT promoter mutation detection relies on obtaining tumor tissue through surgery or biopsy for genetic sequencing, a method with numerous limitations. First, invasive procedures increase patient risk and economic burden. Second, gliomas exhibit significant spatial heterogeneity, and single‐point sampling may not reflect the molecular characteristics of the entire tumor. Third, lesions in certain anatomical locations are difficult to safely access for tissue sampling. Finally, genetic testing is time‐consuming and may delay treatment initiation [9]. Therefore, developing preoperative noninvasive methods for predicting TERT promoter mutation status holds significant clinical value. Magnetic resonance imaging, as the preferred imaging examination for glioma diagnosis and treatment evaluation, not only provides anatomical information about tumors but also reflects tumor biological characteristics through advanced functional imaging techniques. In recent years, the rapid development of radiomics has provided novel solutions to this need.

Radiomics, through high‐throughput extraction and analysis of quantitative features from medical images, can associate imaging phenotypes with tumor genotypes, providing imaging biomarkers for precision diagnosis and individualized treatment of tumors [10]. Multiple studies have confirmed that MRI‐based radiomics methods can effectively predict molecular biomarker status including IDH mutation, 1p/19q codeletion, and MGMT promoter methylation in gliomas, with prediction accuracies ranging from 70% to 95% [11, 12]. Machine learning algorithms such as support vector machines, random forests, and logistic regression are widely applied in radiomics model construction, as these algorithms can identify predictive patterns associated with molecular biomarkers from numerous imaging features [13]. However, previous studies primarily focused on conventional MRI sequences or single functional imaging techniques, with relatively limited research on integrating multiparametric functional imaging information. Diffusion kurtosis imaging and dynamic contrast‐enhanced MRI, as two important advanced functional imaging techniques, provide complementary information about tumors from microstructural and vascular functional perspectives. Their combined application may improve the accuracy of molecular biomarker prediction [14].

Diffusion kurtosis imaging is an advanced non‐Gaussian diffusion imaging technique that reflects tissue microstructural complexity and heterogeneity by quantifying the degree to which water molecule diffusion deviates from a Gaussian distribution. Compared to conventional diffusion tensor imaging, DKI can more accurately describe the true diffusion behavior of water molecules within tumor tissue. Its main parameters include mean kurtosis (MK), mean diffusivity (MD), axial kurtosis, and radial kurtosis. Previous studies have demonstrated that DKI parameters are closely correlated with glioma cell density, proliferative activity, and histological grade, with higher grade gliomas exhibiting significantly higher diffusion kurtosis values than lower grade tumors. Dynamic contrast‐enhanced MRI continuously and dynamically acquires signal changes during contrast agent passage through tumor vasculature and, combined with pharmacokinetic models, quantitatively evaluates tumor vascular permeability and perfusion characteristics. Its main parameters, such as volume transfer constant (Ktrans) and extravascular extracellular space volume fraction (Ve), can reflect the functional status of tumor neovascularization and the degree of blood–brain barrier disruption. The combination of these two techniques is expected to reveal imaging features associated with TERT mutations from different perspectives.

Although a few studies have explored the feasibility of predicting TERT promoter mutations in gliomas using radiomics methods, these studies were primarily based on conventional MRI sequences or single diffusion‐weighted imaging, with room for improvement in predictive performance and lacking in‐depth mechanistic interpretation of predictions. Currently, no studies have systematically evaluated the value of combined DKI and DCE‐MRI radiomics models in predicting TERT promoter mutations. Therefore, this study is aimed at constructing a multiparametric radiomics model based on DKI and DCE‐MRI, investigating its clinical application value in preoperative noninvasive prediction of TERT promoter mutation status in gliomas, and elucidating imaging phenotypic characteristics associated with TERT mutations by analyzing the biological significance of DKI and DCE‐MRI parameters, providing novel technical approaches for preoperative molecular classification and diagnosis of gliomas.

## 2. Materials and Methods

### 2.1. Study Population

This retrospective study enrolled patients with gliomas who underwent surgical treatment in the Department of Neurosurgery at the institution between January 2020 and June 2025. Inclusion criteria were as follows: (1) preoperative complete magnetic resonance examination including conventional MRI sequences, DKI, and DCE‐MRI sequences, (2) postoperative histopathological confirmation of glioma with completed TERT promoter mutation testing, (3) interval between MRI examination and surgery not exceeding 2 weeks, and (4) complete clinical and imaging data. Exclusion criteria were as follows: (1) MRI images with significant motion artifacts or other factors affecting image quality, (2) preoperative receipt of radiotherapy, chemotherapy, or other antitumor treatments, (3) excessively small tumor volume (maximum diameter < 1.0 cm) precluding reliable radiomics analysis, and (4) the presence of other intracranial space‐occupying lesions. A total of 126 patients were ultimately enrolled, including 72 males and 54 females, with an age range of 22–75 years and a median age of 51 years. According to the World Health Organization Classification of Tumors of the Central Nervous System and molecular pathological testing results, 58 cases were TERT promoter mutant, and 68 were wild‐type. Patients were randomly divided into training (*n* = 88; 40 mutant, 48 wild‐type) and validation (*n* = 38; 18 mutant, 20 wild‐type) cohorts at a 7:3 ratio using a random number table. This study was approved by the hospital ethics committee with waiver of informed consent.

### 2.2. MRI Acquisition Protocol

All MRI examinations were performed on a 3.0T magnetic resonance scanner (Discovery MR750, GE Healthcare, Milwaukee, Wisconsin, United States) using a 32‐channel head phased‐array coil. Conventional MRI sequences included axial T1‐weighted imaging, T2‐weighted (T2WI) imaging, fluid‐attenuated inversion recovery (FLAIR) sequence, and contrast‐enhanced T1‐weighted (T1CE) imaging. DKI acquisition employed a single‐shot echo‐planar imaging sequence with the following parameters: repetition time 8500 ms, echo time 95 ms, field of view 240 × 240 mm, matrix 128 × 128, slice thickness 5 mm, slice gap 1 mm, 30 diffusion‐encoding gradient directions, and *b*‐values of 0, 1000, and 2000 s/mm^2^. DCE‐MRI acquisition utilized a three‐dimensional fast spoiled gradient echo sequence with the following parameters: repetition time 5.2 ms, echo time 2.4 ms, flip angle 15°, field of view 240 × 240 mm, matrix 256 × 256, slice thickness 5 mm, and temporal resolution 5.5 s, with 60 phases acquired. Gadopentetate dimeglumine contrast agent (Gd‐DTPA, Magnevist, Bayer Schering Pharma, Berlin, Germany) was injected via antecubital vein at a dose of 0.1 mmol/kg body weight and an injection rate of 3.0 mL/s, beginning at the fourth phase, followed by 20 mL saline flush at the same rate.

### 2.3. Image Postprocessing and Parametric Map Generation

DKI raw images were imported into a Functool postprocessing workstation (GE Healthcare) for analysis. Apparent diffusion coefficient was calculated using monoexponential model fitting, and diffusion kurtosis model fitting generated parametric maps including MK, MD, axial kurtosis, radial kurtosis, axial diffusivity, and radial diffusivity. DCE‐MRI raw images underwent pharmacokinetic analysis using Omni‐Kinetics software (GE Healthcare). The arterial input function was first selected at the internal carotid artery level, followed by pixel‐level fitting based on the extended Tofts model to generate parametric maps of volume transfer constant, extravascular extracellular space volume fraction, rate constant, and plasma volume fraction. All parametric map generation was completed by a radiologist with 8 years of neuroimaging diagnostic experience who was blinded to clinical information and pathological results.

### 2.4. Tumor Segmentation and Region‐of‐Interest Delineation

Tumor segmentation was performed using ITK‐SNAP software (Version 3.8.0) by two radiologists (with 10 and 12 years of experience), who were blinded to clinical and pathological outcomes. To ensure accurate delineation across the heterogeneous enhancement patterns of gliomas, a multisequence, stratified approach was employed. First, all images, including T1CE, T2WI, FLAIR, and the parametric maps derived from DKI (e.g., MK and MD) and DCE‐MRI (e.g., Ktrans and Ve), were rigidly coregistered and fused. This allowed for simultaneous visualization of all sequences during delineation.

The delineation strategy was then stratified based on the tumor′s enhancement pattern:

1. For enhancing tumors (typically high‐grade gliomas): The primary reference for delineating the solid tumor component was the enhancing area on T1CE. T2WI and FLAIR images were used as adjuncts to confidently exclude intratumoral necrotic, cystic, and hemorrhagic regions.
2. For nonenhancing or minimally enhancing tumors (typically low‐grade gliomas): Tumor boundaries were primarily defined by areas of hyperintensity on T2WI and FLAIR, which are characteristics of infiltrative low‐grade tumors. The parametric maps were simultaneously referenced to confirm regions with abnormal diffusion or perfusion, ensuring that the delineated volume accurately represented the tumor extent.
3. For mixed‐pattern tumors: The entire tumor volume, encompassing both enhancing and nonenhancing components, was delineated to comprehensively reflect the tumor′s molecular landscape.

After completion, the three‐dimensional regions of interest were mapped onto the corresponding DKI and DCE‐MRI parametric maps for feature extraction. All delineations were subsequently reviewed by a third senior radiologist to ensure consistency and adherence to the protocol.

### 2.5. Radiomics Feature Extraction

Radiomics feature extraction was completed using the PyRadiomics package (Version 3.0.1) in a Python 3.8 environment. Prior to feature extraction, all images underwent standardized preprocessing, including resampling to 1 × 1 × 1 mm isotropic voxels, signal inhomogeneity correction using the N4 bias field correction algorithm, and normalization of image gray values to a distribution with a mean of 0 and a standard deviation of 1 using Z‐score standardization. Seven major categories of radiomics features were extracted from regions of interest in each parametric map, including first‐order statistics, shape features, gray‐level co‐occurrence matrix features, gray‐level run length matrix features, gray‐level size zone matrix features, gray‐level dependence matrix features, and neighboring gray tone difference matrix features. First‐order statistics described the distribution characteristics of voxel gray values within regions of interest, containing 18 parameters including mean, standard deviation, skewness, kurtosis, energy, and entropy. Shape features described three‐dimensional geometric morphological characteristics of tumors, containing 14 parameters including volume, surface area, sphericity, and compactness. Texture features reflected spatial distribution patterns and heterogeneity characteristics of gray values within regions of interest. To enhance feature robustness, higher order features filtered by wavelet transform were also extracted from original images, employing eight wavelet transform combinations (HHH, HHL, HLH, HLL, LHH, LHL, LLH, and LLL). The entire process of radiomics feature extraction, preprocessing, and feature verification in this study follows the Image Biomarker Standardization Initiative international guidelines [15]. Considering that DKI includes six parametric maps and DCE‐MRI includes four parametric maps, with 154 features extracted from each parametric map, a total of 1540 original radiomics features were extracted. Combined with two clinical features (age and sex), 1542 features were ultimately obtained for subsequent analysis.

### 2.6. Feature Selection and Model Construction

Prior to feature selection, we assessed the reproducibility of radiomics features using ICC analysis based on the 30 randomly selected patients with duplicate segmentations. Features with interobserver ICC < 0.75 were considered unstable and were excluded from subsequent analysis, as this threshold is commonly used to indicate good reliability in radiomics studies. Following this reproducibility screening, 1248 features (81.0%) were retained for further feature selection.

Feature selection was performed separately for radiomics features and clinical features, given their fundamentally different data types, distributions, and biological interpretations.

For radiomics features, we employed a three‐step selection strategy in the training cohort. First, univariate analysis using Mann–Whitney *U* test was performed to compare differences between TERT promoter mutant and wild‐type groups for each radiomics feature, with features having *p* < 0.05 selected for the next step. Second, Spearman correlation coefficient analysis was used to evaluate interfeature correlations among the retained radiomics features. When the absolute correlation coefficient between two features exceeded 0.9, the feature with a stronger association with TERT mutation status (based on the absolute value of the standardized mean difference) was retained, while the redundant feature was removed. Finally, least absolute shrinkage and selection operator regression with 10‐fold cross‐validation was applied to the remaining radiomics features for final dimensionality reduction and selection. The optimal regularization parameter *λ* was determined as the value minimizing cross‐validation error.

For clinical features, we performed separate univariate analysis using appropriate statistical tests based on variable type: independent samples *t*‐test for age (after confirming normal distribution with the Shapiro–Wilk test), chi‐square test for sex (dichotomous nominal variable), and chi‐square test for tumor location (categorical variable). Clinical features with *p* < 0.05 in univariate analysis were considered potentially associated with TERT mutation status and were candidates for inclusion in the integrated model.

After completing feature selection separately for radiomics and clinical features, we constructed four types of models using logistic regression: (1) DKI‐based radiomics model using only selected DKI features, (2) DCE‐MRI‐based radiomics model using only selected DCE‐MRI features, (3) combined radiomics model using selected features from both DKI and DCE‐MRI, and (4) integrated clinical‐radiomics model incorporating both selected radiomics features and significant clinical features. For the integrated model, we entered all selected radiomics features and significant clinical features into a multivariable logistic regression model with backward stepwise selection based on Akaike information criterion to identify the final combination of predictors. Radiomics scores and clinical‐radiomics scores were calculated for each patient based on the linear combination of selected features weighted by their logistic regression coefficients. Additionally, a quantitative parameter model was constructed using only the original DKI and DCE‐MRI parameters (MK, MD, Ka, Kr, AD, RD, Ktrans, Ve, Kep, Vp) without radiomics features, following the same logistic regression procedure.

### 2.7. Molecular Pathological Testing

All surgical specimens were fixed in 10% neutral formalin, paraffin‐embedded, sectioned, and stained with hematoxylin and eosin. Diagnosis was performed by two neuropathologists with more than 15 years of experience according to the 2021 World Health Organization Classification of Tumors of the Central Nervous System. TERT promoter mutation detection employed the Sanger sequencing method, targeting the two hotspot mutation sites TERT promoter C228T and C250T. Genomic DNA was extracted from paraffin‐embedded tissue, target fragments were amplified using polymerase chain reaction, and amplification products underwent bidirectional sequencing after purification. Sequencing results were compared with reference sequences to determine mutation status. The presence of a mutation at either the C228T or C250T site was classified as TERT promoter mutant type, while wild‐type sequences at both sites were classified as wild‐type.

### 2.8. Statistical Analysis

All statistical analyses were completed using R software (Version 4.2.1) and Python 3.8. Continuous variables were expressed as mean ± standard deviation or median, and categorical variables as frequency and percentage. The Shapiro–Wilk test was used to assess data normality. Independent samples *t*‐test was used to compare intergroup differences for continuous variables conforming to normal distribution, Mann–Whitney *U* test for nonnormally distributed variables, and chi‐square test or Fisher′s exact test for categorical variables. Receiver operating characteristic curve analysis was used to evaluate the predictive performance of each model, calculating the area under the curve with 95% confidence intervals and recording sensitivity, specificity, accuracy, positive predictive value, and negative predictive value corresponding to optimal cutoff values. The DeLong test was used to compare differences in the area under the curve among different models. Decision curve analysis was performed to assess the clinical net benefit of models. Calibration curves and the Hosmer–Lemeshow test were used to evaluate model calibration. *p* values less than 0.05 were considered statistically significant, and all tests were two‐sided.

## 3. Results

### 3.1. Patient Characteristics

A total of 126 patients with histologically confirmed gliomas were enrolled in this study, comprising 58 cases with TERT promoter mutations and 68 cases with wild‐type TERT promoter. The training cohort included 88 patients (40 with mutations and 48 wild‐type), while the validation cohort consisted of 38 patients (18 with mutations and 20 wild‐type). The baseline clinical and pathological characteristics of patients in both cohorts are summarized in Table 1.

**Table 1:** Clinical and pathological characteristics of patients in training and validation cohorts.

Characteristic	Training cohort (*n* = 88)		*p* value	Validation cohort (*n* = 38)		*p* value
	TERT‐mutant (*n* = 40)	TERT‐wildtype (*n* = 48)		TERT‐mutant (*n* = 18)	TERT‐wildtype (*n* = 20)	
Age (years)	54.3 ± 11.7	47.6 ± 13.2	0.012	53.8 ± 10.9	48.4 ± 12.8	0.174
Sex (male/female)	24/16	28/20	0.892	11/7	12/8	0.923

Tumor location			0.456			0.631
Frontal lobe	16 (40.0%)	22 (45.8%)		7 (38.9%)	10 (50.0%)	
Temporal lobe	13 (32.5%)	14 (29.2%)		6 (33.3%)	5 (25.0%)	
Parietal lobe	7 (17.5%)	8 (16.7%)		3 (16.7%)	4 (20.0%)	
Occipital lobe	4 (10.0%)	4 (8.3%)		2 (11.1%)	1 (5.0%)	

WHO grade			< 0.001			0.002
Grade 2	6 (15.0%)	22 (45.8%)		3 (16.7%)	9 (45.0%)	
Grade 3	11 (27.5%)	15 (31.3%)		5 (27.8%)	7 (35.0%)	
Grade 4	23 (57.5%)	11 (22.9%)		10 (55.6%)	4 (20.0%)	

IDH status			< 0.001			< 0.001
IDH‐mutant	8 (20.0%)	35 (72.9%)		4 (22.2%)	14 (70.0%)	
IDH‐wildtype	32 (80.0%)	13 (27.1%)		14 (77.8%)	6 (30.0%)	
1p/19q codeletion			0.041			0.089
Codeleted	3 (7.5%)	12 (25.0%)		1 (5.6%)	5 (25.0%)	
Noncodeleted	37 (92.5%)	36 (75.0%)		17 (94.4%)	15 (75.0%)	

### 3.2. Clinical Feature Analysis

Univariate analysis of clinical features using appropriate statistical tests revealed that age was significantly associated with TERT promoter mutation status in the training cohort (*t*‐test, *p* = 0.012), with mutant patients being significantly older (mean 54.3 ± 11.7 years) than wild‐type patients (47.6 ± 13.2 years). Sex showed no significant association with TERT mutation status (chi‐square test, *p* = 0.892), with similar male/female distributions in mutant (24/16) and wild‐type (28/20) groups. Tumor location also showed no significant association (chi‐square test, *p* = 0.456). Based on these results, age was retained as the only clinical feature for subsequent integrated model construction.

### 3.3. Radiomics Feature Selection

From the initial 1,540 radiomics features, 1,248 features were retained after reproducibility screening (ICC ≥ 0.75). Among these, 487 features showed significant differences between TERT promoter mutant and wild‐type gliomas in univariate analysis (Mann‐Whitney U test, P < 0.05). After removing redundant features using Spearman correlation analysis with a threshold of 0.9, 156 radiomics features remained. LASSO regression with 10‐fold cross‐validation identified the optimal lambda value of 0.0342, yielding 12 radiomics features with nonzero coefficients, comprising five features derived from DKI parameters and seven from DCE‐MRI parameters (Table 2). Specifically, the DKI‐derived features included the wavelet‐LHL entropy of MK, gray‐level run length matrix long‐run emphasis of radial kurtosis, gray‐level co‐occurrence matrix contrast of axial kurtosis, first‐order 90th percentile of MD, and wavelet‐HHH skewness of radial diffusivity. The DCE‐MRI‐derived features comprised the gray‐level size zone matrix large area emphasis of Ktrans, first‐order entropy of Ktrans, wavelet‐LHH correlation of Ve, first‐order skewness of Ve, neighboring gray tone difference matrix coarseness of Kep, gray‐level dependence matrix dependence entropy of Vp, and first‐order median of Vp. The feature selection process and the coefficients of selected features are illustrated in Figure 1.

Figure 1Feature selection using LASSO regression. (a) LASSO coefficient profiles of the 156 candidate features. (b) Ten‐fold cross‐validation curve for tuning parameter selection. (c) Coefficients of the 12 selected features in the combined model.

**Figure:**
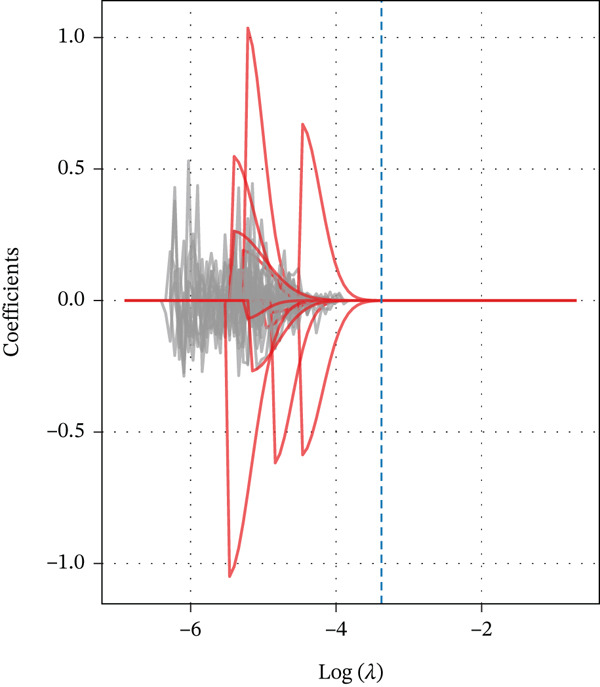
(a)

**Figure:**
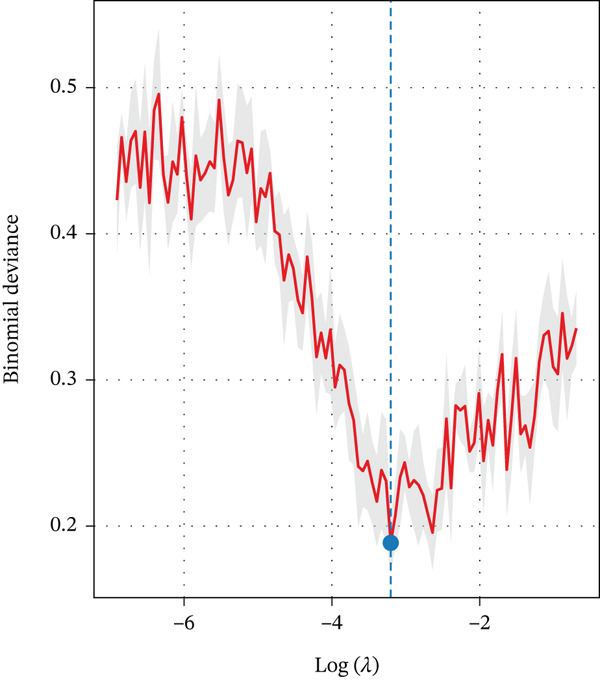
(b)

**Figure:**
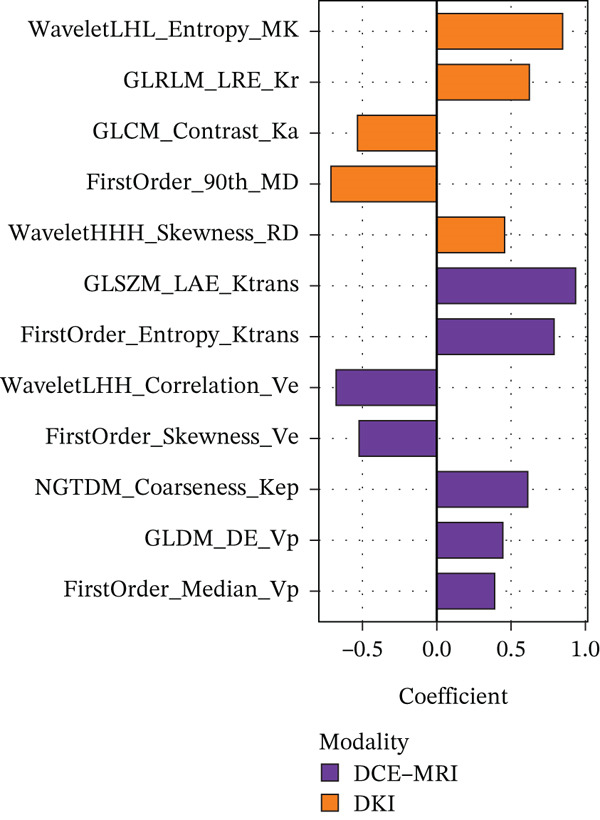
(c)

### 3.4. Integrated Clinical‐Radiomics Model

When the significant clinical feature (age) was combined with the 12 selected radiomics features in a multivariable logistic regression model with backward stepwise selection, age did not retain statistical significance (*p* = 0.234 in the final model) and was eliminated based on the Akaike information criterion. The final integrated model therefore consisted of the same 12 radiomics features as the combined radiomics model. The AUC of the integrated clinical‐radiomics model was 0.963 (95% CI: 0.929–0.997) in the training cohort and 0.944 (95% CI: 0.872–1.000) in the validation cohort, nearly identical to the radiomics‐only combined model (0.961 and 0.943, respectively). The DeLong test showed no significant difference between the two models (*p* = 0.672 in training, *p* = 0.713 in validation), indicating that the addition of clinical age did not provide incremental value beyond the radiomics features. Therefore, we retained the radiomics‐only combined model as our final model for simplicity and to avoid unnecessary complexity.

### 3.5. Comparison of DKI and DCE‐MRI Parameters Between Groups

The quantitative analysis revealed significant differences in multiple DKI and DCE‐MRI parameters between TERT promoter mutant and wild‐type gliomas, as shown in Table 3. In the training cohort, TERT promoter mutant gliomas exhibited significantly higher MK values compared to wild‐type tumors (0.947 ± 0.168 vs. 0.798 ± 0.152, *p* < 0.001), indicating increased tissue complexity and heterogeneity in mutant tumors. Conversely, MD values were significantly lower in mutant gliomas (1.124 ± 0.247 vs. 1.368 ± 0.283 × 10^−3^ mm^2^/s, *p* < 0.001), suggesting more restricted water diffusion. Similar trends were observed for axial kurtosis and radial kurtosis parameters. Regarding DCE‐MRI parameters, TERT promoter mutant gliomas demonstrated significantly higher Ktrans values (0.186 ± 0.094 vs. 0.127 ± 0.068 min^−1^, *p* = 0.002), indicating increased vascular permeability. The Ve values were also elevated in mutant tumors (0.423 ± 0.118 vs. 0.346 ± 0.102, *p* = 0.003), reflecting expanded extravascular extracellular space. Ke*p* values showed significant differences between groups (0.512 ± 0.176 vs. 0.398 ± 0.147 min^−1^, *p* = 0.001), while Vp values did not reach statistical significance. These findings were largely consistent in the validation cohort, confirming the reproducibility of the observed parameter differences.

**Table 2:** Comparison of DKI and DCE‐MRI parameters between TERT promoter mutant and wild‐type gliomas.

Parameter	Training cohort		*p* value	Validation cohort		*p* value
	TERT‐mutant (*n* = 40)	TERT‐wildtype (*n* = 48)		TERT‐mutant (*n* = 18)	TERT‐wildtype (*n* = 20)	
DKI parameters						
MK (mean)	0.947 ± 0.168	0.798 ± 0.152	< 0.001	0.926 ± 0.183	0.812 ± 0.167	0.042
MD (×10^−3^ mm^2^/s)	1.124 ± 0.247	1.368 ± 0.283	< 0.001	1.156 ± 0.271	1.342 ± 0.296	0.037
Ka (mean)	1.082 ± 0.193	0.891 ± 0.174	< 0.001	1.067 ± 0.208	0.914 ± 0.189	0.018
Kr (mean)	0.834 ± 0.157	0.692 ± 0.141	< 0.001	0.817 ± 0.172	0.718 ± 0.153	0.063
AD (×10^−3^ mm^2^/s)	1.387 ± 0.298	1.612 ± 0.334	0.001	1.403 ± 0.312	1.584 ± 0.347	0.088
RD (×10^−3^ mm^2^/s)	0.978 ± 0.223	1.196 ± 0.261	< 0.001	1.002 ± 0.241	1.173 ± 0.278	0.041
DCE‐MRI parameters						
Ktrans (min^−1^)	0.186 ± 0.094	0.127 ± 0.068	0.002	0.178 ± 0.087	0.134 ± 0.073	0.089
Ve	0.423 ± 0.118	0.346 ± 0.102	0.003	0.408 ± 0.126	0.358 ± 0.114	0.197
Kep (min^−1^)	0.512 ± 0.176	0.398 ± 0.147	0.001	0.497 ± 0.183	0.412 ± 0.162	0.126
Vp	0.047 ± 0.023	0.041 ± 0.019	0.176	0.044 ± 0.021	0.043 ± 0.020	0.872

Abbreviations: AD, axial diffusivity; Ka, axial kurtosis; Kep, rate constant; Kr, radial kurtosis; Ktrans, volume transfer constant; MD, mean diffusivity; MK, mean kurtosis; RD, radial diffusivity; Ve, extravascular extracellular volume fraction; Vp, plasma volume fraction.

### 3.6. Diagnostic Performance of Radiomics Models

The diagnostic performance of the DKI‐based, DCE‐MRI‐based, and combined radiomics models for predicting TERT promoter mutation status is presented in Table 2. In the training cohort, the DKI‐based radiomics model achieved an area under the curve of 0.847 with 95% confidence interval of 0.768–0.926, demonstrating good discriminative ability. The model showed a sensitivity of 77.5%, a specificity of 81.3%, an accuracy of 79.5%, a positive predictive value of 77.5%, and a negative predictive value of 81.3% at the optimal cutoff value. The DCE‐MRI‐based radiomics model exhibited superior performance compared to the DKI model, with an AUC of 0.892, a sensitivity of 82.5%, a specificity of 85.4%, and an accuracy of 84.1%. The combined radiomics model, which integrated features from both DKI and DCE‐MRI, achieved the highest diagnostic performance in the training cohort with an AUC of 0.961, a sensitivity of 92.5%, a specificity of 91.7%, and an accuracy of 92.0%.

**Table 3:** Diagnostic performance of radiomics models for predicting TERT promoter mutation status.

Model	Cohort	AUC (95% CI)	Sensitivity (%)	Specificity (%)	Accuracy (%)	PPV (%)	NPV (%)
DKI‐based	Training	0.847 (0.768–0.926)	77.5	81.3	79.5	77.5	81.3
	Validation	0.823 (0.691–0.955)	72.2	85.0	78.9	76.5	81.8
DCE‐MRI‐based	Training	0.892 (0.821–0.963)	82.5	85.4	84.1	82.5	85.4
	Validation	0.869 (0.752–0.986)	83.3	85.0	84.2	83.3	85.0
Combined	Training	0.961 (0.927–0.995)	92.5	91.7	92.0	90.2	93.6
	Validation	0.943 (0.871–1.000)	88.9	95.0	92.1	94.1	90.5

Abbreviations: AUC, area under the curve; CI, confidence interval; NPV, negative predictive value; PPV, positive predictive value.

In the validation cohort, the performance metrics remained robust, although slightly decreased compared to the training cohort, which is expected due to the smaller sample size. The DKI‐based model yielded an AUC of 0.823; the DCE‐MRI‐based model achieved an AUC of 0.869, while the combined model maintained excellent performance with an AUC of 0.943. The combined model demonstrated a sensitivity of 88.9%, a specificity of 95.0%, an accuracy of 92.1%, a positive predictive value of 94.1%, and a negative predictive value of 90.5% in the validation cohort. The receiver operating characteristic curves for all three models in both training and validation cohorts are displayed in Figure 2.

Figure 2Receiver operating characteristic curves of the three radiomics models for predicting TERT promoter mutation status. (a) Training cohort. (b) Validation cohort.

**Figure:**
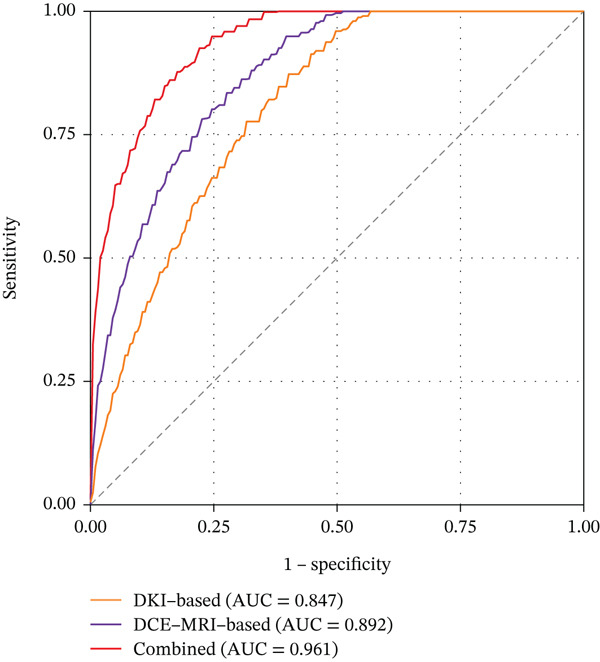
(a)

**Figure:**
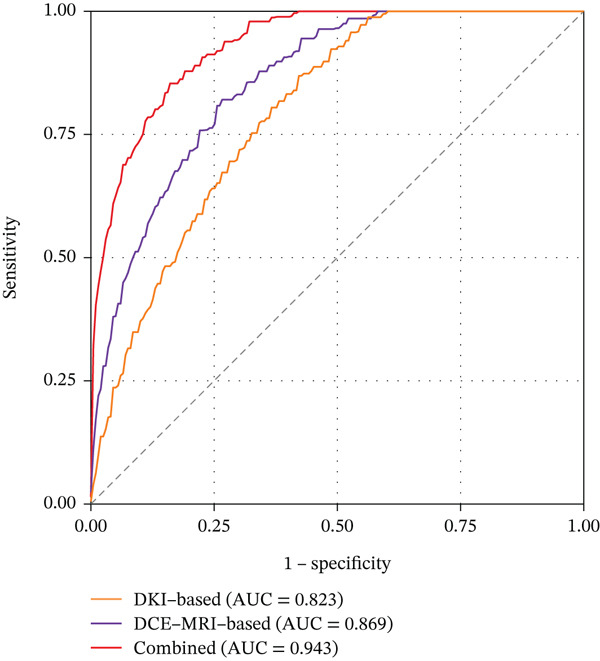
(b)

### 3.7. Performance of the Quantitative Parameter Model

To assess the incremental value of radiomics features over conventional quantitative imaging parameters, we constructed a quantitative parameter model using logistic regression based on the 10 core DKI and DCE‐MRI parameters (MK, MD, Ka, Kr, AD, RD, Ktrans, Ve, Kep, and Vp). After backward stepwise selection based on the Akaike information criterion, five parameters were retained in the final model: MK, MD, Ktrans, Ve, and Kep.

In the training cohort, the quantitative parameter model achieved an AUC of 0.812 (95% CI: 0.734–0.890), with a sensitivity of 75.0%, a specificity of 77.1%, and an accuracy of 76.1%. In the validation cohort, the model demonstrated an AUC of 0.783 (95% CI: 0.648–0.918), with corresponding sensitivity, specificity, and accuracy of 72.2%, 75.0%, and 73.7%, respectively.

### 3.8. Comparison of Model Performance

The DeLong test was performed to compare the diagnostic performance among the three radiomics models. As shown in Table 4, the combined radiomics model and the DCE‐MRI‐based radiomics model demonstrated significantly superior performance compared to the quantitative parameter model in both the training (*p* < 0.001 and *p* = 0.008, respectively) and validation (*p* < 0.001 and *p* = 0.021, respectively) cohorts. The DKI‐based radiomics model showed numerically higher AUC values than the quantitative parameter model in both the training (0.847 vs. 0.812) and validation (0.823 vs. 0.783) cohorts; however, this difference did not reach statistical significance (*p* = 0.087 in the training cohort; *p* = 0.134 in the validation cohort).

**Table 4:** Comparison of AUC values among conventional MRI, quantitative parameter, and radiomics models.

Comparison	Training cohort		Validation cohort	
	**Difference in AUC**	**p value**	**Difference in AUC**	**p value**
Combined vs. quantitative parameter model	0.149	< 0.001	0.160	< 0.001
Combined vs. conventional MRI model	0.163	< 0.001	0.182	< 0.001
Combined vs. DKI‐based model	0.114	0.003	0.120	0.006
Combined vs. DCE‐MRI‐based model	0.069	0.012	0.074	0.048
DCE‐MRI‐based vs. quantitative parameter model	0.080	0.008	0.086	0.021
DKI‐based vs. quantitative parameter model	0.035	0.087	0.040	0.134
DCE‐MRI‐based vs. DKI‐based model	0.045	0.038	0.046	0.052

### 3.9. Clinical Utility Assessment

Decision curve analysis was conducted to evaluate the clinical utility of the three radiomics models, as illustrated in Figure 3. The analysis demonstrated that all three models provided net benefit across a wide range of threshold probabilities from 0.1 to 0.9 in both training and validation cohorts. The combined model consistently showed the highest net benefit compared to the treat‐all and treat‐none strategies, as well as the single‐modality models. In the validation cohort, the combined model maintained superior net benefit across threshold probabilities ranging from 0.15 to 0.85, indicating its potential clinical applicability. At a threshold probability of 0.5, which represents a balanced consideration of false positives and false negatives, the combined model achieved a net benefit of 0.83 in the training cohort and 0.76 in the validation cohort, substantially higher than the single‐modality models.

Figure 3Decision curve analysis for the three radiomics models. (a) Training cohort. (b) Validation cohort. The *y*‐axis represents the net benefit, and the *x*‐axis represents the threshold probability. The gray line represents the assumption that all patients have TERT promoter mutations, and the black line represents the assumption that no patients have mutations.

**Figure:**
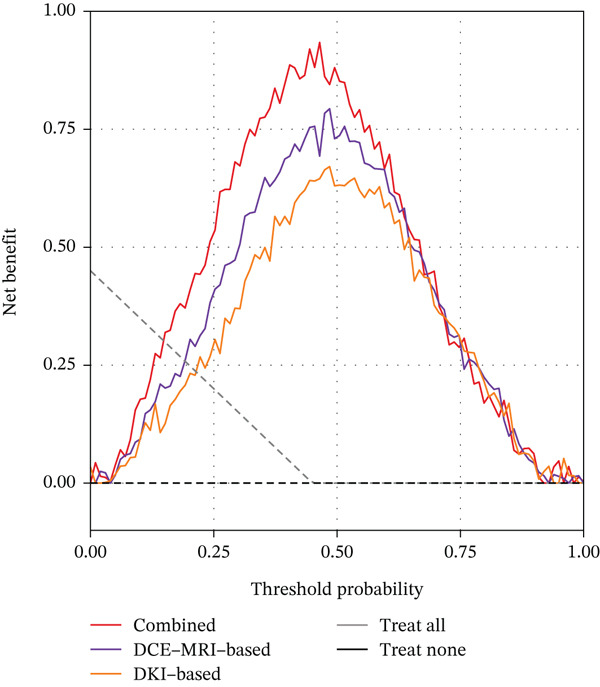
(a)

**Figure:**
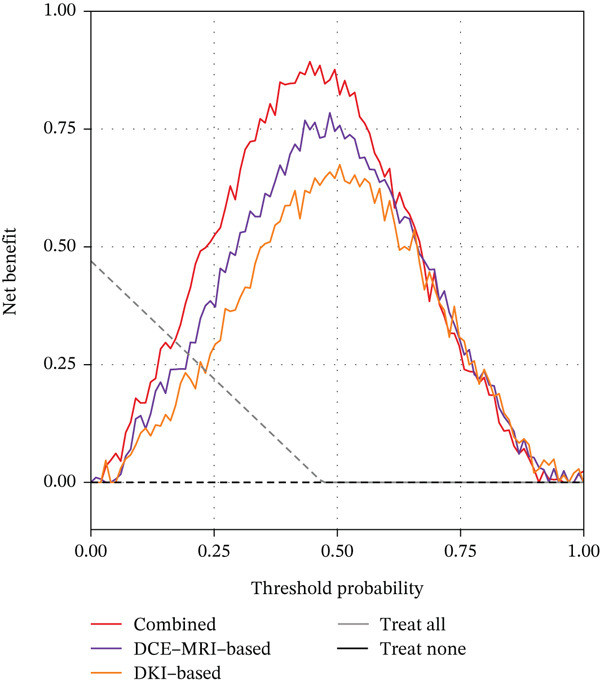
(b)

The calibration curves demonstrated good agreement between predicted probabilities and actual outcomes for all three models in both cohorts, as shown in Figure 4. The Hosmer–Lemeshow test yielded nonsignificant *p* values for the combined model in both training (*p* = 0.438) and validation (*p* = 0.612) cohorts, indicating satisfactory calibration. The calibration slope for the combined model was 0.94 in the training cohort and 0.89 in the validation cohort, suggesting minimal overestimation or underestimation of risk. The calibration intercept was close to zero in both cohorts, further confirming the reliability of the predicted probabilities. These results indicate that the combined radiomics model not only achieves high discriminative ability but also provides well‐calibrated probability estimates for TERT promoter mutation status.

Figure 4Calibration curves of the three radiomics models. (a) DKI‐based model. (b) DCE‐MRI‐based model. (c) Combined model. The diagonal dashed line represents perfect prediction, and the solid line represents the observed performance of each model.

**Figure:**
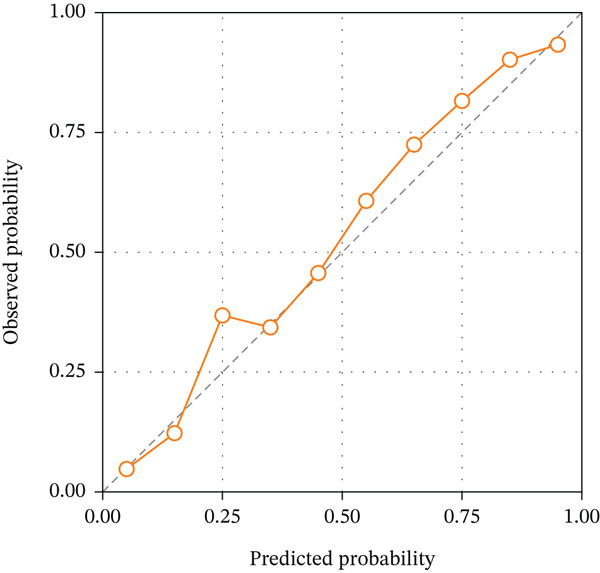
(a)

**Figure:**
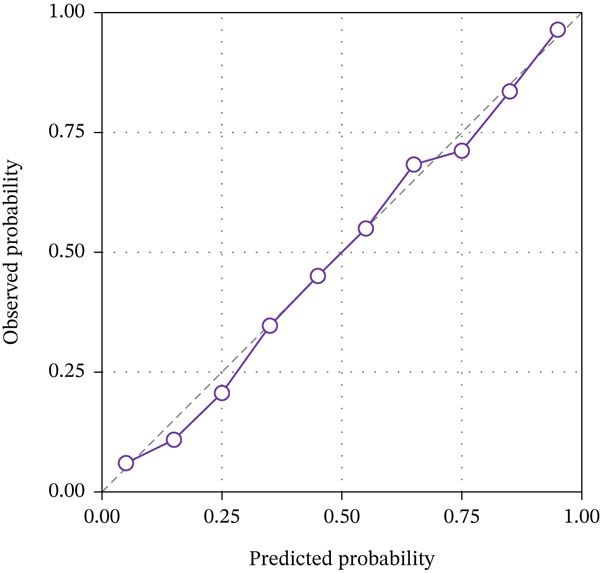
(b)

**Figure:**
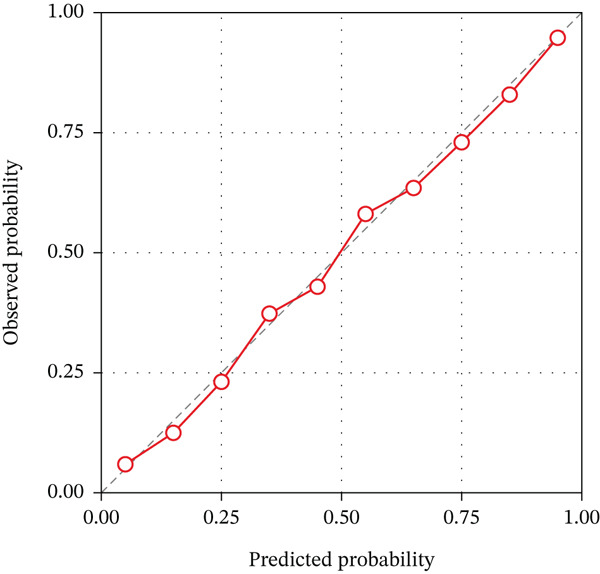
(c)

### 3.10. Subgroup Analysis by Tumor Grade

A subgroup analysis was performed to evaluate the predictive performance of the combined radiomics model across different WHO grades, as presented in Table 5. In WHO Grade 2–3 gliomas, the combined model achieved an AUC of 0.924 in the training cohort and 0.897 in the validation cohort, with accuracy rates of 88.2% and 83.3%, respectively. For WHO Grade 4 gliomas, the model demonstrated an AUC of 0.952 in the training cohort and 0.936 in the validation cohort, with slightly higher accuracy rates of 91.2% and 92.9%, respectively. Although the performance appeared numerically higher in high‐grade gliomas, the differences did not reach statistical significance (*p* = 0.287 in training cohort, *p* = 0.412 in validation cohort), suggesting that the combined model maintains robust predictive ability across different tumor grades. This finding indicates the potential broad applicability of the radiomics approach regardless of histological grade.

**Table 5:** Performance of the combined radiomics model in different WHO grades.

WHO grade	Cohort	AUC (95% CI)	Sensitivity (%)	Specificity (%)	Accuracy (%)
Grades 2–3	Training	0.924 (0.854–0.994)	88.2	88.1	88.2
	Validation	0.897 (0.772–1.000)	81.3	85.0	83.3
Grade 4	Training	0.952 (0.898–1.000)	94.1	89.5	91.2
	Validation	0.936 (0.838–1.000)	90.0	94.2	92.9

We further evaluated the performance of the combined radiomics model stratified by IDH mutation status. As shown in Table 6, the combined model demonstrated excellent predictive performance in both IDH‐mutant and IDH‐wildtype subgroups. In the training cohort, the model achieved AUCs of 0.932 (95% CI: 0.876–0.988) and 0.951 (95% CI: 0.891–1.000) for IDH‐mutant and IDH‐wildtype subgroups, respectively, with no significant difference between subgroups (DeLong test, *p* = 0.432). Similar results were observed in the validation cohort (AUC: 0.908 vs. 0.936, *p* = 0.521). These findings indicate that the combined radiomics model maintains robust predictive ability regardless of IDH status, further supporting its generalizability across different molecular subtypes of gliomas.

**Table 6:** Performance of the combined radiomics model stratified by IDH status.

IDH status	Cohort	AUC (95% CI)	Sensitivity (%)	Specificity (%)	Accuracy (%)	PPV (%)
IDH‐mutant	Training (*n* = 53)	0.932 (0.876–0.988)	88.9	85.7	86.8	84.2
IDH‐wildtype	Training (*n* = 35)	0.951 (0.891–1.000)	90.6	92.3	91.4	96.7
IDH‐mutant	Validation (*n* = 18)	0.908 (0.812–1.000)	80.0	92.3	88.9	88.9
IDH‐wildtype	Validation (*n* = 20)	0.936 (0.846–1.000)	92.9	83.3	90.0	92.9

Abbreviations: AUC, area under the curve; CI, confidence interval; PPV, positive predictive value.

## 4. Discussion

This study constructed a multiparametric radiomics model based on diffusion kurtosis imaging and dynamic contrast‐enhanced MRI for preoperative prediction of TERT promoter mutation status in gliomas. The results demonstrated that the combined radiomics model exhibited excellent predictive performance in both training and validation cohorts, with areas under the curve reaching 0.961 and 0.943, respectively, significantly superior to single‐modality models.

TERT promoter mutation represents one of the most common molecular genetic alterations in gliomas, with occurrence frequency reaching up to 80% in glioblastomas [16]. The two hotspot mutation sites, TERT promoter C228T and C250T, create novel ETS transcription factor binding sites, leading to upregulation of TERT gene expression and enhanced telomerase activity, thereby conferring unlimited proliferative capacity and immortalization characteristics to tumor cells [17]. The prognostic significance of TERT promoter mutations demonstrates heterogeneity across different molecular subtypes of gliomas. In IDH‐wildtype glioblastomas, TERT promoter mutations are associated with worse prognosis, whereas in IDH‐mutant lower grade gliomas, they may indicate relatively favorable prognosis [18, 19]. This complex prognostic association underscores the importance of accurately identifying TERT promoter mutation status for precision stratified management of gliomas. Traditional molecular detection methods require obtaining tumor tissue through surgery or biopsy, with limitations including high invasiveness, sampling errors, and high time costs [20]. Therefore, developing imaging‐based noninvasive prediction methods holds significant clinical value.

This study found that TERT promoter mutant gliomas exhibited significant characteristics in DKI parameters, with mutant tumors demonstrating significantly higher MK values than wild‐type tumors, while MD values were markedly lower. The pathophysiological basis of this phenomenon can be explained at the microstructural level. DKI reflects tissue microstructural complexity and heterogeneity by quantifying the degree to which water molecule diffusion deviates from a Gaussian distribution [21]. Higher diffusion kurtosis values reflect greater restriction of water molecule diffusion within tissue, which is closely related to pathological features of TERT‐mutant gliomas. TERT promoter mutations promote unlimited tumor cell proliferation by maintaining telomere length, leading to increased cell density, decreased extracellular space, and more compact and complex tissue structure [22]. Furthermore, TERT‐mutant tumors are often accompanied by more active neovascularization and microvascular proliferation. These chaotic vascular structures further increase microenvironmental heterogeneity and impede free diffusion of water molecules, thereby resulting in elevated diffusion kurtosis values [23]. Previous studies have confirmed significant correlations between DKI parameters and tumor cell density and Ki‐67 proliferation index, with higher grade gliomas exhibiting significantly higher diffusion kurtosis values than lower grade tumors [24, 25]. The findings of this study further extend the application value of DKI in glioma molecular classification, suggesting that DKI not only reflects tumor histological grade but also captures microstructural features associated with specific molecular alterations.

DCE‐MRI parameters in this study similarly demonstrated good predictive value. TERT promoter mutant gliomas exhibited significantly elevated volume transfer constant and extravascular extracellular space volume fraction, revealing intrinsic connections between TERT mutations and tumor vascular characteristics. TERT function is not limited to classical telomere length maintenance roles. Increasing evidence indicates that TERT possesses telomere‐independent noncanonical functions, including promoting angiogenesis, enhancing cell proliferation, and inhibiting apoptosis [26]. TERT can promote neovascularization through upregulation of vascular endothelial growth factor expression, while TERT‐mutant tumors are often accompanied by higher microvascular density and more disorganized vascular structures [27]. These neovessels are predominantly immature vessels composed of incomplete endothelial cells and basement membranes, with significantly increased vascular permeability [28]. Volume transfer constant (Ktrans) is a comprehensive parameter reflecting vascular permeability and perfusion, with its elevation directly reflecting the degree of tumor vascular barrier disruption [29]. The increase in extravascular extracellular space volume fraction (Ve) suggests greater contrast agent leakage into the extracellular space, which is related to increased vascular permeability and extracellular matrix remodeling in TERT‐mutant tumors [30]. Notably, the DCE‐MRI radiomics model in this study demonstrated superior predictive performance compared to the DKI model, possibly reflecting that TERT mutation effects on tumor vascular function are more pronounced than microstructural alterations.

Radiomics methods, through high‐throughput extraction and analysis of quantitative features from medical images, can capture subtle texture and heterogeneity information imperceptible to the human eye, providing powerful tools for precision tumor phenotyping [31]. This study extracted 1540 radiomics features from DKI and DCE‐MRI parametric maps, encompassing shape, first‐order statistics, and multiple texture features. Through LASSO regression, 12 optimal predictive features were selected to construct the combined model. These features primarily included higher order texture features based on wavelet transformation and first‐order statistical features, quantifying tumor internal spatial heterogeneity patterns from different perspectives. Gray‐level co‐occurrence matrix contrast and gray‐level run length matrix features reflected spatial distribution patterns of pixel gray values within tumors, while entropy features quantified randomness and disorder of pixel intensity distributions [32]. TERT‐mutant gliomas, due to their higher cell proliferative activity, more complex vascular structures, and more prominent necrotic regions, exhibit greater spatial heterogeneity, which these radiomics features can sensitively capture. The combined model, by integrating complementary information from DKI and DCE‐MRI, simultaneously reflected tumor microstructural characteristics and vascular functional properties, thereby demonstrating optimal predictive performance. Notably, while the DKI‐based radiomics model showed numerically higher AUC values compared to the quantitative parameter model, this difference did not reach statistical significance. This finding suggests that the additional texture and heterogeneity information captured by DKI radiomics features may provide only modest incremental value over conventional DKI parameters alone. In contrast, the DCE‐MRI‐based radiomics model significantly outperformed its quantitative counterpart, indicating that radiomics analysis may be particularly valuable for extracting predictive information from perfusion imaging. This differential pattern may reflect the greater complexity and heterogeneity of vascular characteristics associated with TERT mutations, which are more effectively captured by texture‐based features than by global parameter averages.

The innovation of this study lies in combining DKI and DCE‐MRI, two advanced functional imaging techniques, for predicting TERT promoter mutation status in gliomas and employing radiomics methods for in‐depth feature mining. Compared to previous studies, this study possesses the following advantages: First, a more comprehensive multiparametric imaging strategy was adopted. DKI reflects tissue microstructural complexity, while DCE‐MRI provides vascular functional information. Their combination characterizes tumor pathophysiological features from different dimensions [33]. Second, this study enrolled a relatively large sample size with rigorous division into training and validation cohorts, fully validating model generalizability. Third, standardized feature extraction procedures and robust feature selection methods were employed, ensuring reproducibility of study results. However, previous studies primarily focused on conventional MRI sequences or single functional imaging techniques, with relatively limited predictive performance [34, 35]. For example, Tian et al. constructed a TERT mutation prediction model based on conventional MRI sequences with an AUC of only 0.78, while studies employing multiparametric imaging and deep learning methods, although achieving good results, often lacked biological interpretation of prediction mechanisms [36]. This study, by analyzing the biological significance of DKI and DCE‐MRI parameters, provided a solid pathophysiological foundation for prediction models.

Subgroup analysis in this study showed that the combined radiomics model maintained good predictive performance across different WHO grades of gliomas, suggesting favorable generalizability of the model. However, notably, prediction accuracy was slightly higher in high‐grade gliomas compared to low‐grade tumors, possibly related to more prominent imaging features in high‐grade gliomas. Decision curve analysis further confirmed the practical value of the combined model in clinical decision‐making. Across a wide range of threshold probabilities, the model provided net benefit to clinicians, assisting in formulating individualized treatment plans. For example, for patients predicted to have TERT‐mutant status, clinicians may consider more aggressive treatment strategies or enrollment in clinical trials targeting telomerase [37]. Additionally, radiomics scores can serve as risk stratification tools, identifying high‐risk patient populations and providing a basis for follow‐up monitoring and treatment adjustment.

This study has several limitations that warrant improvement in future work. First, this was a single‐center retrospective study. Although internal validation was performed, external independent cohort validation is lacking, and model extrapolation capacity requires further confirmation in multicenter prospective studies. Different magnetic resonance scanners and imaging parameters may lead to variability in radiomics features. Standardized imaging protocols and feature extraction procedures are crucial for clinical translation of models [15]. Second, this study only enrolled patients who underwent a complete multiparametric MRI examination preoperatively, potentially introducing selection bias. Future studies should expand sample size and include more glioma patients with different subtypes and stages to improve model representativeness. Third, this study employed region‐of‐interest‐based radiomics analysis. Although necrotic and cystic areas were carefully avoided, manual delineation may still have subjectivity. Future exploration of deep learning–based automatic segmentation methods could improve analysis objectivity and efficiency [38]. Finally, this study primarily focused on TERT promoter mutation as a single molecular biomarker, whereas glioma molecular classification involves synergistic effects of multiple genes, such as IDH, MGMT, and 1p/19q. Future studies should explore the radiomics potential in simultaneously predicting multiple molecular biomarkers to construct more comprehensive molecular phenotype prediction systems [39].

## 5. Conclusion

In conclusion, this study successfully constructed a multiparametric radiomics model based on DKI and DCE‐MRI for preoperative prediction of TERT promoter mutation status in gliomas. The combined model demonstrated excellent predictive performance and clinical utility value. DKI parameter‐reflected tissue microstructural complexity and DCE‐MRI parameter‐revealed vascular functional abnormalities provided a pathophysiological foundation for understanding the imaging phenotype of TERT mutations. Radiomics methods, through high‐throughput feature extraction and machine learning algorithms, achieved precise quantification of tumor heterogeneity. This study provides novel technical approaches for preoperative noninvasive molecular classification of gliomas and is expected to play important roles in clinical decision‐making, prognostic assessment, and individualized treatment. Future studies should further validate model robustness in multicenter large‐sample cohorts and explore application potential in predicting treatment response and survival prognosis.

## Author Contributions

**Peng Du**, **Aihong Cao**, **Yinjiao Wang:** conceptualization. **Song Gao**, **Shenao Zhang**, **Lang Chen**, **Peng Du:** methodology. **Song Gao**, **Shenao Zhang**, **Lang Chen:** software/simulation. **Song Gao**, **Shenao Zhang**, **Lang Chen:** validation/formal analysis. **Yinjiao Wang**, **Aihong Cao:** data curation, clinical oversight. **Song Gao**, **Shenao Zhang:** visualization. **Song Gao**, **Shenao Zhang:** writing – original draft. **Song Gao**, **Shenao Zhang**, **Yinjiao Wang**, **Lang Chen**, **Aihong Cao**, **Peng Du:** writing – review and editing. **Peng Du**, **Aihong Cao:** supervision. **Peng Du**, **:** funding acquisition.**Song Gao**, **Shenao Zhang**, and **Yinjiao Wang** contributed equally to this work and are considered co‐first authors.

## Funding

This work was supported by the Research Project of Jiangsu Provincial Health Commission (Z2024021), the Xuzhou Medical University Affiliated Hospital Development Fund (XYFY202460), and the Clinical Technology Key Personnel Advanced Training Program of Xuzhou (2025GG020).

## Ethics Statement

The study protocol was approved by the Institutional Review Board of The Second Affiliated Hospital of Xuzhou Medical University (Approval Number: 2025021701). All procedures were conducted in accordance with relevant guidelines and regulations and with the Declaration of Helsinki. This retrospective study used data collected as part of participants′ routine clinical care. Written informed consent for participation was waived in accordance with national legislation and institutional requirements.

## Consent

The authors have nothing to report.

## Conflicts of Interest

The authors declare no conflicts of interest.

## Data Availability

Simulation code, configuration files (LGG/HGG parameter sets), and analysis scripts will be made publicly available upon publication (e.g., GitHub/Zenodo DOI). During peer review, access will be provided to editors/reviewers upon request to the corresponding authors. Synthetic imaging outputs and derived masks are available on reasonable request; deidentified derived metrics can be provided.
